# Progressive Cutaneous Cholesterol Emboli Syndrome

**DOI:** 10.7759/cureus.78773

**Published:** 2025-02-09

**Authors:** Elizabeth S Szumel, Samuel A Mickel-Hulse, Michael Contarino

**Affiliations:** 1 Internal Medicine, University of North Carolina at Chapel Hill, Chapel Hill, USA; 2 Internal Medicine-Pediatrics, University of North Carolina at Chapel Hill, Chapel Hill, USA

**Keywords:** arterial occlusion, atherosclerotic plaque, cardiac catheterization complication, cholesterol crystals, complications of cholesterol emboli syndrome, digital ischemia, ischemic cerebrovascular disease, risk of anticoagulation, vascular disease

## Abstract

Cholesterol emboli syndrome is a rare syndrome of tissue ischemia and necrosis caused by the embolization of cholesterol crystals from atherosclerotic plaques, leading to vascular occlusion. This report documents a case of cholesterol emboli syndrome in a 72-year-old male with multiple cardiovascular risk factors including end-stage renal disease, atrial fibrillation, hypertension, hyperlipidemia, and type 2 diabetes. We describe this patient’s atypical presentation with upper extremity rather than lower extremity digital ischemia as a presenting sign and significant subsequent functional decline exacerbated by his comorbidities. In addition, we review the available treatment options for cholesterol emboli syndrome, including medical management, and discuss the risks of surgical intervention, including death and limb loss. This study concludes that atypical manifestations of cholesterol emboli syndrome can result in particularly morbid outcomes due to loss of fine motor skills in their hands, reducing patients’ independence and exacerbating other medical conditions.

## Introduction

Cholesterol emboli syndrome (also known as cholesterol crystal embolism [CES]) is a rare syndrome of tissue ischemia and necrosis caused by embolization of cholesterol crystals from atherosclerotic plaques, leading to vascular occlusion [1,2]. The prevalence of clinically significant CES has not been well documented and varies based on the clinical scenario but has been reported at 0.02-2.9% in high-risk study populations including those who have recently undergone heart catheterization and those with known significant atherosclerotic disease [1,3-7]. The true prevalence of CES is suspected to be higher, noted to be 77% in patient status post-abdominal aortic aneurysm resection based on autopsy studies. Importantly, not all cases are thought to cause clinically significant disease [1,8]. Risk factors for CES are similar to those for atherosclerotic disease, such as male genetic sex, older age, tobacco use, diabetes mellitus, hypertension, and elevated serum cholesterol levels, in addition to recent intravascular procedures or cardiovascular surgery [1,2,8]. CES is caused when atherosclerotic plaques, usually within larger arteries such as the aorta, rupture. Cholesterol crystals contained within the plaques are then released into the arterial system as micro-emboli and lodge in small and medium arteries distal to the primary plaque. These crystals trigger inflammation and platelet activation, which can worsen vascular occlusion and contribute to tissue hypoxia and cell death [1,2]. CES can affect different organs and, in some cases, correlates with the location of the originating atheromatous plaque [8]. The abdominal aorta and the ilio-femoral arteries are the most common sources of atheroembolisum; thus, cholesterol crystals most commonly affect tissue distal to these arteries, such as the kidneys, lower extremities, and the mesentery, leading to renal failure, skin findings such as blue toe syndrome or livedo reticularis, and/or mesenteric ischemia [1,8].

In this report, we present a case of CES in a patient with an unusually large burden of digital ischemia of the hands.

## Case presentation

A 72-year-old male presented to the emergency department with bilateral discoloration of his fingertips. The patient’s finger discoloration was first noted one month prior, when he had been hospitalized for a syncopal episode thought to be related to recent discontinuation of midodrine. Midodrine had been previously prescribed to treat hypotension during the patient’s nightly peritoneal dialysis (PD) sessions. During his prior hospitalization, CTA showed no acute occlusion of the arteries of his upper extremities, and he had pulses present on Doppler. At the time of discharge, there was suspicion that the patient may have Raynaud’s phenomenon. Since discharge from his previous hospitalization, the patient’s fingers had become more discolored and increasingly painful, and he had developed new blisters. The patient had no history of trauma to the fingers. The patient was seen by his primary care physician for hospital follow-up one week before presenting to the emergency department and was awaiting an appointment with vascular surgery. However, the continued progression of the discoloration prompted him to present to the emergency department for more urgent evaluation.

Past medical history was notable for end-stage renal disease (ESRD) on PD, atrial fibrillation on apixaban, hypertension, hyperlipidemia, and type two diabetes. He was a former smoker but quit in his 30s and denied other drug use. The patient underwent a left cardiac catheterization four months prior to presenting to the emergency department with access via the right groin. This catheterization was complicated by a dissection flap and occlusion of the common femoral artery. The patient subsequently underwent a right femoral endarterectomy with patch angioplasty and percutaneous transluminal angioplasty of the right popliteal artery with drug-coated angioplasty three months prior to presentation.

**Figure 1:**
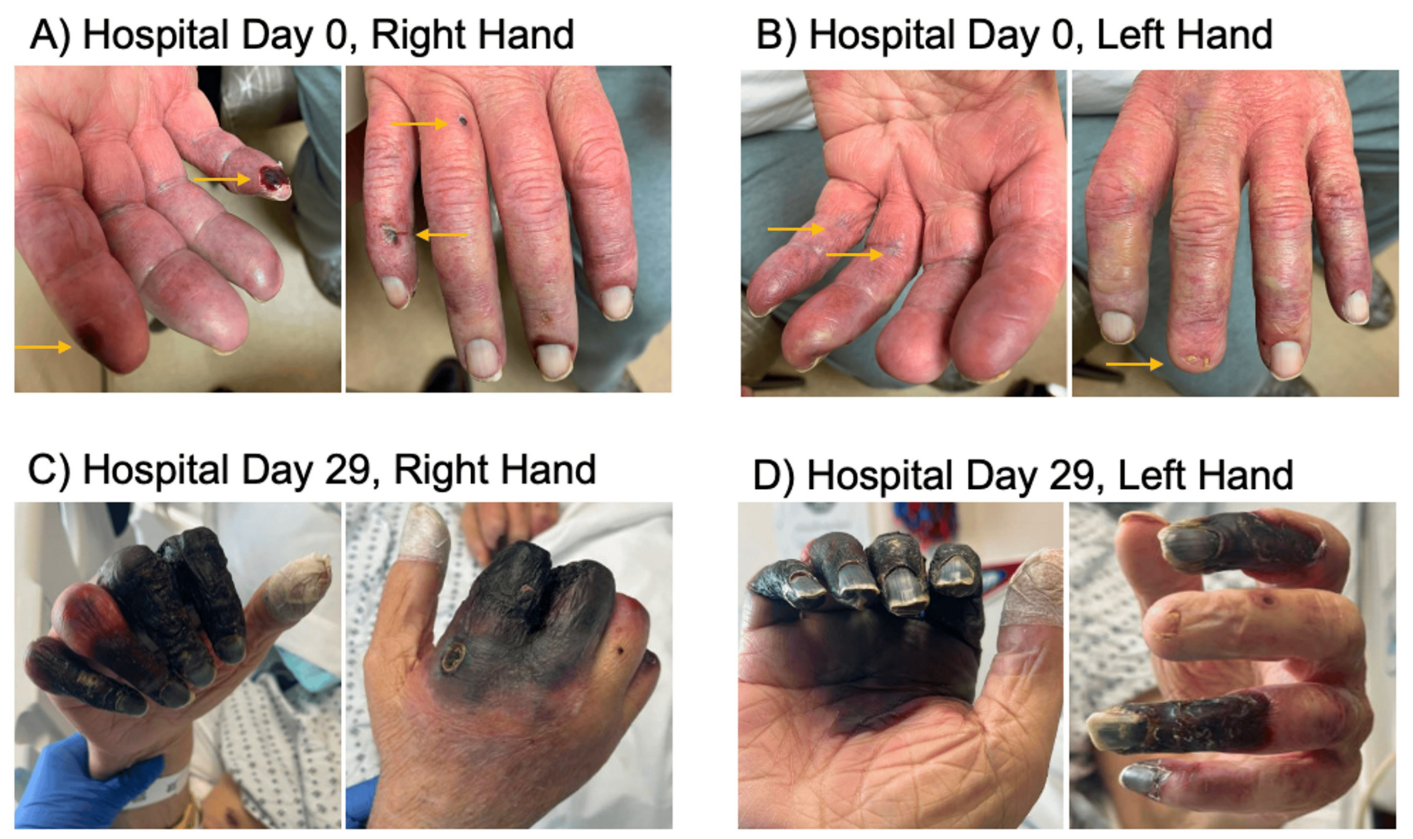
Progression of cholesterol emboli syndrome Photos demonstrate the progression of digital ischemia in a patient with cholesterol emboli syndrome within a 29-day period. (A, B) Arrows indicate the initial lesions seen on hospital day 0. (C, D) Progression of ischemia by hospital day 29 in the right and left hands.

On physical examination, there was discoloration of all five fingers on the right and left hands with necrosis of multiple fingertips (Figure 1). The discoloration started at the proximal interphalangeal joint. Discoloration of the toes on the left foot was also noted. The patient had palpable radial and ulnar pulses bilaterally and a palpable dorsalis pedis pulse on the left foot.

At the time of admission, the differential included small vessel vasculitis (including anti-neutrophil cytoplasmic autoantibody [ANCA] vasculitis, immune complex small-vessel vasculitis, anti-glomerular basement membrane [GBM], and cryoglobulinemia), Raynaud’s phenomenon or syndrome, peripheral vascular disease, and arterial emboli. An infectious workup and autoimmune workup were both sent on admission, which were notable for elevated C-reactive protein (710 mg/L) and erythrocyte sedimentation rate (94 mm/hr), positive antinuclear antibody with elevated complement 4 (49.8 mg/dL), negative ANCA, rheumatoid factor, and immunoglobulins. Additionally, during the hospitalization, he had two sets of blood cultures collected, both of which were negative. Upper extremity arterial duplexes were negative for any obstruction. Hand films were not concerning for acute osteomyelitis. CT head without contrast was notable for hypoattenuation in the periventricular white matter concerning for small vessel ischemic disease. Echocardiogram was only significant for evidence of moderate pulmonary hypertension, with no valvular disease or masses and a normal ejection fraction.

On admission, Rheumatology, Vascular Surgery, and Hematology were consulted. Despite the broad differential, Rheumatology pointed out that a thromboembolic etiology must be considered given his recent cardiac catheterization and recommended consulting Dermatology for a biopsy. Dermatology was primarily concerned for cholesterol emboli syndrome given the patient’s cardiac history, recent cardiac procedures, recent initiation of anticoagulation, diabetes, and hypertension, as well as his borderline elevated eosinophils. Skin biopsy revealed lenticular spaces in the arterial lumen interrupted by pineal eosinophilic material, which was diagnostic of CES. CTA of the chest identified diffuse moderate calcified and noncalcified atherosclerotic plaque of the thoracic aorta and origin of the great vessels.

When CES was diagnosed, the patient’s dose of atorvastatin was increased to 80 mg for maximal secondary prevention in the setting of the patient's known atherosclerotic cardiovascular disease. The patient was continued on apixaban throughout their hospitalization because of their history of atrial fibrillation, and clopidogrel was continued to prevent clot formation after their recent femoral endarterectomy. Orthopedic surgery was consulted to discuss the definitive management of necrotic tissue. They determined that the patient’s digits would need to be monitored until the necrosis stopped progressing. Only at that point would it be safe to amputate the affected digits. Amputation too soon would result in poor wound healing, increased risk for infection, increased pain, and multiple surgeries. The patient did not need to remain in the hospital and could follow up with Orthopedic Surgery outpatient for monitoring of the necrosis. Palliative care was consulted for assistance with pain management. Pain was treated with a combination of acetaminophen, gabapentin, and oral and IV opioids.

Discharge was prolonged due to dialysis discussions, as the patient could no longer perform PD at home secondary to the progressing necrosis of his fingers. The patient’s hospital stay was complicated by deconditioning, constipation in the setting of opioid use, development of a scrotal lesion that required antibiotics, and poor nutrition due to his inability to hold utensils, resulting in hyponatremia and hypokalemia. The patient was evaluated by the nutrition team while admitted and was provided with nutritional supplements.

The patient also had periods of altered mental status (AMS) that became more frequent throughout his hospitalization. His AMS was initially attributed to pain requiring frequent opioid medication use. Head CT findings were notable for small-vessel disease, and an MRI of his brain was notable for iron deposition in the caudate and putamen along with white matter changes. These findings were not consistent with an acute embolic event. Contrasted studies were not performed due to the patient's ESRD. Ultimately, the patient's mental status improved somewhat prior to discharge, and his AMS was thought to be multifactorial in the setting of toxic metabolic encephalopathy from opiates and possibly uremia, hospital-acquired delirium, malnutrition, and potential central nervous system (CNS) involvement of CES.

The patient was discharged home with a fentanyl patch, hydromorphone as needed, and duloxetine. He had support from his wife, a friend, and several family members who had been trained to perform PD. Orthopedic surgery attempted to arrange follow-up with the patient, but multiple follow-up visits were canceled for unknown reasons. The patient ultimately passed away approximately two months after discharge.

## Discussion

This report discusses the case of a 72-year-old male who presented with progressive cutaneous cholesterol emboli syndrome in the digits of his upper and lower extremities. The patient had numerous risk factors for CES, including recent cardiac catheterization, diabetes, hypertension, and hyperlipidemia. This case is unusual in that this patient presented with predominantly upper extremity lesions rather than lower. While upper extremity lesions have been reported, foot and toe lesions are more frequently reported as a presenting symptom and usually correlate with ruptured plaques in more distal large arteries such as the abdominal aorta or ilio-femoral arteries [1,8-14]. This patient’s upper extremity lesions are consistent with emboli from plaque at a less commonly identified source: his thoracic aorta and origin of the great vessels [1,8]. This patient’s presentation was also interesting due to potential CNS involvement with syncope as a presenting symptom during his previous hospitalization, worsening mental status changes throughout hospitalization, and ischemic changes seen on brain imaging. Stroke and CNS sequelae have been reported in other cases of CES but are less common [4,8,15]. These potential CNS complications would also be consistent with this patient’s proximal plaque.

The patient’s CES was treated with a high-intensity statin, continuation of clopidogrel and apixaban, multimodal pain control, and watchful waiting for the necrosis to demarcate before amputation. High-intensity statins are universally recommended treatment for CES to prevent further embolization [1,2,8]. Though there are less data to support it, antiplatelet agents are also commonly used as treatment to prevent clot formation. In this patient’s case, he had additional indications with recent vascular stenting and therefore was continued on clopidogrel [2,8].

Steroids were discussed as a potential treatment; however, they were ultimately not used due to concerns for worsened hyperglycemia with the patient’s diabetes. Several case reports suggest the utility of steroids particularly for cutaneous and renal manifestations of CES; however, these benefits have not been studied more rigorously [13-18]. Despite this, it may have been worth reconsidering a course-limited trial of steroids for this patient given the fact that he could have been closely monitored during his prolonged hospital stay.

The patient was on apixaban at presentation for anticoagulation in the setting of atrial fibrillation. This was continued throughout his treatment for CES. Anticoagulation has been proposed as a risk factor for CES as there is an association between initiation of anticoagulation and CES; it has been proposed that anticoagulation could increase the likelihood of plaque rupture [1,2,8]. However, the limited studies investigating this association have not demonstrated an increase in risk, and the patient population at risk for CES often overlaps with those needing anticoagulation for other reasons [1,2,8].

Currently, there are few other commonly accepted treatment options for CES, and most of the current treatment options are preventive and supportive as opposed to curative. Other than steroids, less commonly reported treatments for inflammation and emboli burden include iloprost and low-density lipoprotein apheresis; however, research on the effectiveness of these treatments is lacking [18-20]. There is no consensus on a standard treatment regimen for CES, and treatment plans often rely on specialists at the institution where the patient is being treated. For patients with identified plaques, surgical interventions are also an option, though they carry high morbidity and mortality when the plaque is in the suprarenal aorta, as in this case [21]. With this patient’s ESRD and plaque location in the thoracic aorta at the base of the great vessels, he was a poor surgical candidate. Additional research into current and novel treatment options is needed.

Lastly, this case is unique in that it highlights how significant loss of function secondary to CES can affect a patient’s quality of life and influence medical complications. This patient’s loss of hand function due to his progressing lesions prevented him from independently feeding himself, which led to poor nutrition and subsequent electrolyte abnormalities. He was also unable to perform PD independently, and because hemodialysis did not align with his goals of care, significant caregiver support had to be arranged prior to discharge. Had the patient's lesions presented in the lower extremities rather than the upper extremities, he would have still been able to perform PD and activities of daily living that require fine motor skills such as feeding.

While in most cases, patients' lower extremities are affected more by CES than the upper extremities, this potential impact on prognosis and quality of life is an important factor when determining treatment plans and discussing outcomes with patients and their families. Ultimately, treatment of functional limitations may require support from multi-disciplinary teams such as occupational and physical therapy, nutrition, and palliative care.

## Conclusions

Cholesterol emboli syndrome is a rare but morbid complication of cardiovascular procedures including cardiac catheterization. Treatment of cholesterol emboli syndrome is not standardized, and primary treatment regimens currently focus on minimizing extension of the disease with the use of high-intensity statins, with consideration of surgical amputation in specific cases. Antiplatelet agents, anticoagulation, and steroids are also considered on a case-by-case basis. Further study is needed to determine the true efficacy of these interventions as well as novel treatment options; however, opportunity for research is limited predominantly by the rarity of CES.

When the digits of the upper extremities are primarily affected, this can lead to additional complications revolving around a patient’s ability to complete activities of daily living, such as eating, drinking, and, for this patient, operating his PD machine independently. By necessity, treatment of cholesterol emboli syndrome should be individualized for each patient, taking into account the extent and location of disease, comorbid conditions, and goals of care.
